# The Spatial-Temporal Variation Characteristics of Natural Vegetation Drought in the Yangtze River Source Region, China

**DOI:** 10.3390/ijerph18041613

**Published:** 2021-02-08

**Authors:** Jun Yin, Zhe Yuan, Ting Li

**Affiliations:** 1 Department of Geographical Sciences, Faculty of Resources and Environmental Science, Hubei University, Wuhan 430062, China; yinjun19880209@126.com (J.Y.); hubu1997@126.com (T.L.); 2 Changjiang River Scientific Research Institute, Changjiang Water Resources Commission of the Ministry of Water Resources of China, Wuhan 430010, China

**Keywords:** ecological drought, spatial-temporal characteristics, probability and return period, Yangtze River Source Region

## Abstract

In the context of climate change, ecosystem in Yangtze River Source Region (YRSR) is under threat from severe droughts. This study introduced a new natural vegetation drought index, standardized supply-demand water index (SSDI), and identified natural vegetation drought events and parameters (e.g., duration, severity, peak, and coverage area) based on run theory. Then the drought-prone regions were investigated via 2-dimensional joint copula. The results indicate that (1) compared with traditional meteorological drought index, the SSDI is reliable and can reflect the comprehensive characteristics of the ecological drought information more easily and effectively; (2) the YRSR had witnessed the most severe drought episodes in the periods of late-1970s, mid-1980s, and mid-1990s, but the SSDI showed a wetting trend since the mid-2000s. Additionally, droughts in the Southern YRSR were relatively more severe with longer drought duration; (3) in most areas of Togton River Basin and Dam River Basin, the severe ecological drought events occurred more frequently; (4) drought duration and severity in the YRSR were more susceptible to temperature when the temperature rise was above 1.0 °C. The average drought duration and severity increased by 20.7% and 32.6% with a temperature rise of 1 °C. Investigating and evaluating drought characteristics, causes, and drought index effectiveness provide essential information for balanced water resource allocation, utilization, and drought prevention. Understanding these spatial-temporal characteristics of drought and return period was useful for drought risk assessment and sustainable development of water resources.

## 1. Introduction

Drought is one of the widespread climatic extreme events that is characterized by prolonged water deficit. Both social economic systems and ecological environment systems are vulnerable to drought [1,2,3]. According to statistics, about 8% of global vegetated land area has been affected by extreme drought, causing GPP (Gross Primary Productivity) reduction of 1.5 PgC (petagrams of carbon) during 2001–2011 [4]. It has been estimated that the cereal losses due to droughts reached up to 1820 million Mg in worldwide in the past four decades [5]. Globally, more than 11 million deaths have been caused by drought disasters and more than 2 billion people have been affected since the 1990s [6]. Furthermore, the drought frequency and intensity in the mid to late 21st century were projected to increase in the context of climate change [6,7], which would lead to more drought problems in the future. Thus, it is important to assess drought characteristics in the planning and management of water resources. In recent research, a plenty of drought indices have been developed to identify different types of droughts, such as meteorological drought, hydrologic drought, agricultural drought, and socioeconomic drought [8,9]. The most widely used drought indices include standardized precipitation index (SPI) [10,11], Standardized Precipitation-Evapotranspiration Index (SPEI) [12], Palmer drought severity index (PDSI) [13], soil moisture drought index [14], and so on. In addition, a run theory for identifying drought parameters concerning drought duration, magnitude, and intensity was proposed by Yevjevich [15]. This approach has been applied in several drought models and analyses [16,17,18,19,20,21].

However, most drought indices use a single hydro-meteorological variable (e.g., precipitation, runoff) or in combination with other meteorological elements, loosely connected to ground conditions [22]. Although many drought indices are proposed for meteorological drought, hydrologic drought, and agricultural drought, few have been used for terrestrial natural vegetation. The Vegetation Condition Index (VCI) [23] is a kind of remote-sensing index and can be used for vegetation drought assessment. It takes Normalized Difference Vegetation Index (NDVI) as input, which has advantages in describing the vegetation biomass in drought condition. However, it is difficult to be used for drought forecasting compared with the indices with hydro-meteorological variables as input. A natural vegetation drought index with simple hydro-meteorological variables is needed for drought modeling and forecasting. Weng et al. proposed a Generalized Drought Assessment Index (GDAI) for assessing drought events [24]. This index considers water supply and water demand of water resources systems. Similarly, a natural vegetation drought index can be built with water supply and water demand during the growth period. Additionally, these two major inputs can be evaluated by hydro-meteorological variables, making it easy to model and forecast vegetation drought.

The Yangtze River Source Region (YRSR), located in the center of the Tibet Plateau, is one of areas that are sensitive and vulnerable to climate. Since the mid-20th century, the hydro-meteorological variables and water resources showed considerable changes in the YRSR under global warming [25,26]. The temperature, precipitation, actual evapotranspiration, and runoff of the YRSR increased by 0.34 °C/decade, 11.4 mm/decade, 7.6 mm/decade, and 3.3 mm/decade, respectively, in the last 60 years [27]. Furthermore, the annual average temperature and precipitation are projected to increase by 2.2 °C and 9.8% in the next 30 years based on CMIP5 (Coupled Model Intercomparison Project Phase 5) Climate Models [28]. Both the water supply and water demand of natural vegetation (especially grass and forest) has been influenced in these warmer and wetter climate conditions. Investigating natural vegetation drought characteristics in the context of climate change is helpful for freshwater planning and management. Thus, we addressed the following key issues: (1) introduce a new natural vegetation drought index considering the difference between water supply and demand during the growth period; (2) reveal the spatial-temporal variation characterization of natural vegetation drought in the YRSR; (3) investigate the drought-prone regions by bivariate probability and return period.

## 2. Materials and Methods

### 2.1. Study Area

**Figure 1:**
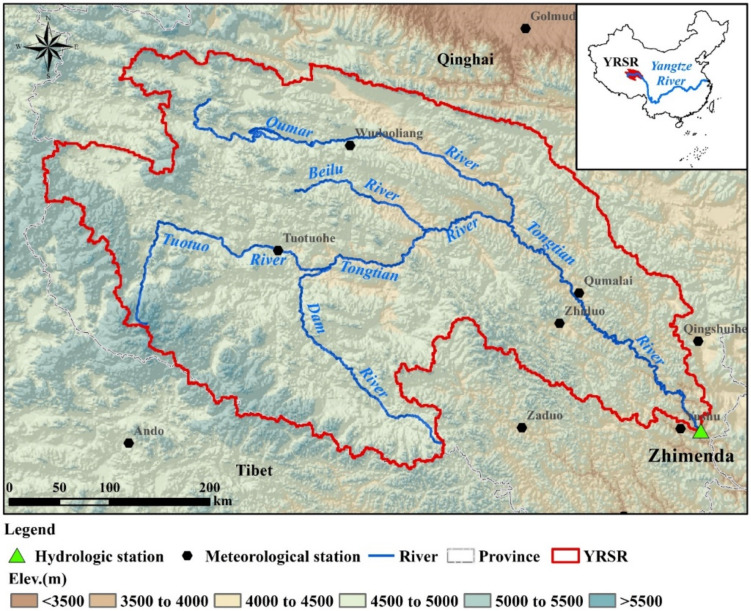
Location of the Yangtze River Source Region (YRSR), China.

Yangtze River Source Region (Latitude: 32°25′ E and 35°53′ E; Longitude: 89°43′ E to 97°19′ E), located in the western Tibetan plateau, covers an area of 159,065 km^2^ (Figure 1). The elevation ranges from 6456 m in the West to 3512 mm in the East, with an average of 4779 m. The average annual precipitation is approximately 327.4 mm, which is concentrated from June to September. The average annual temperature is about −5.5–3.0 °C from northeast to southwest, with an average of 2.9 °C [27,29]. The drought index is 3.67 in the YRSR, which means the climate is very dry. The land cover in the YRSR consists primarily of grasslands (63.8%) and unused land (29.4%). Water area and forest land are less dominant land cover types, accounting for 6.5% and 0.3% of total area of YRSR. Due to the cold and dry climatic conditions, the eco-hydrology process of the whole region is sensitive to climate change.

### 2.2. Datasets

#### 2.2.1. Hydro-Meteorological Data

The daily discharge data measured from 1960 to 2016 at Zhimenda hydrological station were provided by Qinghai Hydrology Bureau. The daily meteorological data, including precipitation, highest temperature, lowest temperature, average temperature, sunshine hours, wind speed, relative humidity over the past 57 years (1960 to 2016) at 12 meteorological stations were obtained from National Meteorological Information Center (http://data.cma.cn/). The distribution of these selected hydro-meteorological stations was shown in Figure 1.

#### 2.2.2. Land Use/Land Cover Data

Land use/Land cover (LULC) data with a spatial resolution of 1 × 1 km for the years 1990, 2000, and 2010 was obtained from Resource and Environment Data Cloud Platform (RESDC) (http://www.resdc.cn/). This dataset is generated by remote sensing images with manual visual interpretation method. The quality has been controlled and integration has been checked by RESDC.

#### 2.2.3. Soil Map and Soil Properties

The digital soil map and properties of dominant soil types were collected from the China soil database (http://www.soil.csdb.cn/), which is provided by Institute of Soil Science, Chinese Academy of Sciences.

#### 2.2.4. Digital Elevation Model (DEM) Data

The DEM data with a resolution of 90 m was downloaded from the CGIARCSI GeoPortal (https://srtm.csi.cgiar.org/).

### 2.3. Methodology

#### 2.3.1. Drought Index and Drought Identification

##### Standardized Water Supply-Demand Index (SSDI)

The SSDI describes the degree of deviation between dry and wet conditions based on the difference between water supply and demand [30]. The algorithm of this index is similar to SPEI, but it defines drought in a broad term, considering water resources shortage instead of climatological factors. Taking crop evapotranspiration and effective precipitation as input, the SSDI has been used to identify the agricultural drought [30]. In the same way, it can be used to quantify the natural vegetation drought conditions. For the vegetation drought estimation in YRSR, this study made several assumptions. Taking into account only perennial or seasonal vegetation and their corresponding phenological stages. As there are hardly any rainfed crops in the Yangtze River Source Region, rainfed crops were not included. According to the previous studies, the green water is important for the natural grass and forest vegetation [31]. Thus, the green water flow (GWF) was selected as the water supply in this study. While, the water demand can be represented by ecological water requirement (EWR) of vegetation. With these two major variables, the SSDI for ecological drought evaluation is briefly described as follows: 

The water surplus or deficit (*WD_i_*) is defined as Equation (1)

(1) $$WD_{i}=GWF_{i}-EWR_{i}$$

where, *GWF_i_* and *EWR_i_* refer to green water flow and natural vegetation water requirement at each station or subregion for the *i*th month respectively. The *GWF* can be quantified by hydrological model. In this study, the ArcSWAT 2012 (https://swat.tamu.edu/software/arcswat) (Blackland Research & Extension Center, Temple, TX, USA) is used for model setup and parameterization and *GWF* estimation [32,33]. In the previous study, we already built a SWAT (Soil and Water Assessment Tool) model for YRSR and simulated the flows of green water, as shown in Yuan et al. [28]. Vegetation type, climate, and soil moisture can all determine the vegetation’s *EWR*, which was calculated by quota valuation mode in this research.

(2) $$EWR=K_{s}\times K_{c}\times ET_{0}$$

where *ET*_0_ represents the potential evapotranspiration of vegetation (mm), which can be calculated by Penman–Monteith formula. *Kc* represents the plant water potential coefficient; *Ks* represents the soil moisture coefficient. The detailed mathematical procedure can be found in Zhao et al. [34].

Accumulated *WD* value in a window of *k*-months (*D^k^*) is calculated as Equation (3).

(3) $$\begin{array}{c} D_{n}^{k}=\overset{k-1}{\underset{i=0}{\sum }}\left(GWF_{n-i}-EWR_{n-i}\right)\;n\geq k \end{array}$$

where, *n* is the analyzed month.

According to SPEI calculation procedure, a three-parameter log-logistic distribution is selected to consider the *D^k^* series:

(4) $$F\left(x\right)={\left[1+{\left(\frac{\alpha }{x-\gamma }\right)}^{\beta }\right]}^{-1}$$

where, *F*(*x*) is the probability distribution function (PDF) of *D^k^*; *α*, *β*, *γ* are the scale, shape, and origin parameters, respectively, which can be estimated by *L*-moment method [35].

Finally, the obtained values of *F*(x) are converted into the standardized values by Equation (5)

(5) $$\begin{array}{l} if\;P\leq 0.5\\ W=\sqrt{-2ln\left(P\right)}\\ SSDI=W-\frac{c_{0}+c_{1}W+c_{2}W_{2}}{1-d_{1}W+d_{2}W_{2}+d_{3}W_{3}}\\ else\\ W=\sqrt{-2ln\left(1-P\right)}\\ SSDI=\frac{c_{0}+c_{1}W+c_{2}W_{2}}{1-d_{1}W+d_{2}W_{2}+d_{3}W_{3}}-W \end{array}$$

where, *P* = 1 − *F*(*x*); according to SPEI calculation procedure, the constants are *C*_0_ = 2.515517, *C*_1_ = 0.802853, *C*_2_ = 0.010328, *d*_1_ = 1.432788, *d*_2_ = 0.189269, and *d*_3_ = 0.001308. Table 1 describes the categorization of drought magnitude by *SSDI*.

##### Drought Event Identification Based on Theory of Runs

**Figure 2:**
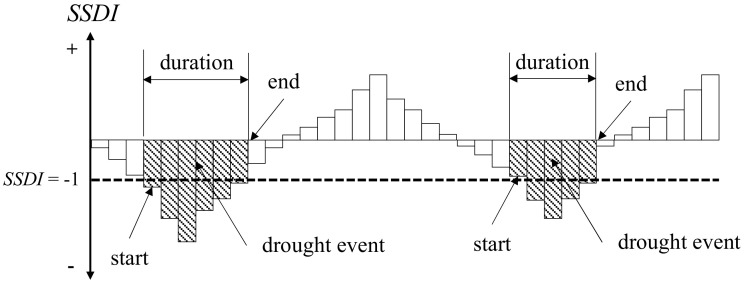
Drought event identification using the run theory for a given threshold level [9].

The ‘run theory’ was proposed for drought characteristics definition by Yevjevich [15], which has been widely used for drought event identification [9,24]. In this study, we suggest that a drought event starts when the value of the *SSDI* value reaches −1.0 or less and ends when the *SSDI* value is more than −1.0 (Figure 2). Then, the characteristics of each drought event can be described by the parameters as listed below:

1. Drought duration (*DD*) is defined as the time between the initiation and termination of a drought event, which is expressed in months in this study.
  (6) $$DD_{n}=Tt_{n}-Ti_{n}$$
  where *DD_n_* refers to drought duration for the *n*th drought event. *Ti_n_* and *Tt_n_* are initiation time and termination time, respectively.

The average duration can be calculated as follows:

(7) $$DD_{avg}=\frac{\sum_{n=1}^{ND}DD_{n}}{ND}$$

where *DD_avg_* refers average duration during a given period. *DD_n_* refers to drought duration for the *n*th drought event. *ND* is the number of drought events during a given period.

2. Drought severity (*DS*) is the sum of *SSDI*s during the drought duration.
  (8) $$DS_{n}=\overset{DD_{n}}{\underset{i=1}{\sum }}SSDI_{i}$$
  where *DS_n_* refers to drought severity for the *n*th drought event.

The average severity can be calculated as follows:

(9) $$DS_{avg}=\frac{\sum_{n=1}^{ND}DS_{n}}{ND}$$

where *DS_avg_* refers to average severity during a given period. *DS_n_* refers to drought severity for the *n*th drought event. *ND* is the number of drought events during a given period.

3. Drought peak (*DP*) is the maximum absolute value of *SSDI*s of a drought event.
  (10) $$DP_{n}=\max\left(\left|SSDI_{Ti_{n}}\right|,\left|SSDI_{Ti_{n}+1}\right|,\cdots ,\left|SSDI_{Tt_{n}-1}\right|,\left|SSDI_{Tt_{n}}\right|\right)$$
  where *DP_n_* refers to drought peak for the *n*th drought event. *Ti_n_* and *Tt_n_* are initiation time and termination time, respectively.

4. Drought coverage area (*DA*) is the region affected by the drought, which is calculated as follows:
  (11) $$DA_{n}=\frac{A\left(n\right)}{A}\times 100\%$$
  where, *DA_n_* refers to the ratio of average drought affected area. *A*(*i*) is the area of region experiencing drought conditions. *A* is the area of the study region. In this research, the parameter *A* represents the area of grassland and forest ecosystem in the YRSR.

#### 2.3.2. Trend Analysis for Drought Characteristic

##### Linear Regression Analysis

To identify the interannual trends of drought parameters (e.g., SSDI values and coverage area), the linear regression method was adopted to eliminate the increase or decrease rate [36], which can be calculated as follows:

Linear regression method was utilized to analyze the interannual trends of drought parameters (e.g., SSDI values and coverage area) [36]. The equation is listed as follows:

(12) $$\theta_{Slope}=\frac{n\times \sum_{i=1}^{n}\left(i\times X_{i}\right)-\sum_{i=1}^{n}i\sum_{i=1}^{n}X_{i}}{n\times \sum_{i=1}^{n}i^{2}-{\left(\sum_{i=1}^{n}i\right)}^{2}}$$

where *θ*_slope_ is the linear slope of the time series variable, which can be used to characterize the increase or decrease rate during a given study period; *n* is the number of years (here *n* = 15); *X_i_* is the drought parameter for the *i*th year (*I* = 1,2,…*n*).

##### Mann–Kendall Test

The Mann–Kendall test is widely used in the field of hydrology and climatology [37]. The Mann–Kendall statistic, *Z_MK_*, can be calculated by the following equation:

(13) $$Z_{MK}=\left\{\begin{array}{c} \frac{S-1}{\sqrt{VAR\left(S\right)}}\;if\;S>0\\ 0\;if\;S=0\\ \frac{S+1}{\sqrt{VAR\left(S\right)}}\;if\;S<0 \end{array}\right.$$

where, Statistic *S* can be calculated through the following equation:

(14) $$S=\overset{n-1}{\underset{k=1}{\sum }}\overset{n}{\underset{j=k+1}{\sum }}sgn\left(X_{j}-X_{k}\right)$$

where, *j* > *k*; *n* is the number of years, *X_j_* and *X_k_* are the annual values in the years *j* and *k*, respectively. The equation sgn(*X_j_* − *X_k_*) can be calculated as follows:

(15) $$sgn\left(X_{j}-X_{k}\right)=\left\{\begin{array}{c} 1\;if\;X_{j}-X_{k}>0\\ 0\;if\;X_{j}-X_{k}=0\\ -1\;if\;X_{j}-X_{k}<0 \end{array}\right.$$

where, *Z_MK_* follows a standard normal distribution, if |*Z*| > *Z*_1__−α∕2_, where α denotes the significance level, then the trend is significant. 

The results of the statistical analysis above can obtained by MATLAB (The MathWorks, Natick, MA, USA).

### 2.3.3. Calculation of Bivariate Probability and Return Period via Copula Function

#### Selection of Marginal Distributions

Previous research has recommended a larger set of marginal distributions for fitting drought variables [38,39,40]. In this study, the exponential, Gamma, Weibull, Generalized Pareto, Generalized Extreme Value distributions were selected as candidates to fit the DD, DS, and DP. The cumulative distribution functions are presented in Equations (16)–(20).

(16) $$\mathrm{Exponential}:\;F\left(x\right)=1-exp\left(-\frac{x-\beta }{\alpha }\right)$$

(17) $$\mathrm{Gamma}:\;F\left(x\right)=\int_{0}^{x}\frac{x^{\alpha -1}}{\beta^{\alpha }\Gamma \left(\alpha \right)}exp\left(-\frac{x}{\beta }\right)dx$$

(18) $$\mathrm{Weibull}:\;F\left(x\right)=1-exp{\left(-\frac{x}{\alpha }\right)}^{\beta }$$

(19) $$\mathrm{Generalized}\;\mathrm{Pareto}:\;F\left(x\right)=1-{\left[1-\beta \left(\frac{x-\lambda }{\alpha }\right)\right]}^{\frac{1}{\beta }}$$

(20) $$\mathrm{Generalized}\;\mathrm{Extreme}\;\mathrm{Value}\;\begin{array}{c} F\left(x\right)=exp\left\{-{\left[1-k\left(\frac{x-\lambda }{\alpha }\right)\right]}^{\frac{1}{\beta }}\right\}\;\left(\beta \neq 0\right) \end{array}$$

where Γ(⋅) is the gamma function. *F(x)* is the marginal distribution and *x* is the value of drought variable. *α*, *β*, *λ* are scale, shape, and location parameters, respectively, which can be estimated by maximum likelihood method.

The fitted distributions were compared against empirical distribution estimated by Equation (21) [41].

(21) $$P_{m}=\frac{m-0.44}{n+0.12}$$

where *P_m_* is the empirical cumulative probability of the *m*th value. m represents *m*th smallest value in the dataset arranged in ascending order. *n* is the total number. 

In this study, the Kolmogorov–Smirnov (K-S) test was used for exploring the suitability of marginal distribution [12]. Additionally, the optimal marginal distribution was selected with Squared Euclidean Distance (SED) [42].

#### Selection of Copulas

Bivariate models based on copula functions were employed for probabilistic modelling and return period estimation in this study. A copula function *C* can link marginal distributions to their bivariate probability distribution as follows:

(22) $$P\left(X\leq x,Y\leq y\right)=F_{XY}\left(x,y\right)=C\left(F_{X}\left(x\right),F_{Y}\left(y\right)\right)$$

where *F_XY_*(*x*,*y*) is the bivariate probability distribution. *F_X_*(·) and *F_Y_*(·) are marginal distributions. *X* and *Y* are correlated variables.

Among all types of copulas, the Archimedean copulas with only one parameter derived by Genest and MacKay [43] have been widely used for analyzing drought characteristics [33,44,45]. In this research, three families of 2-dimensional Archimedean copulas, Clayton, Frank and Gumbel, were used to construct bivariate distributions for *DD* vs. *DS*, *DD* vs. *DP* and *DS* vs. *DP*. Expressions of the mentioned copulas are presented as follows:

(23) $$\mathrm{Clayton}:\;\begin{array}{c} C\left(u,v\right)=max\left({\left(u^{-\theta }+v^{-\theta }-1\right)}^{-\frac{1}{\theta }},0\right)\;\theta \in \left(0,\infty \right) \end{array}$$

(24) $$\mathrm{Frank}:\;\begin{array}{c} C\left(u,v\right)=-\frac{1}{\theta }ln\left(1+\frac{\left(e^{-\theta u}-1\right)\left(e^{-\theta v}-1\right)}{e^{-\theta }-1}\right)\;\theta \in (-\infty ,0)\cap (0,+\infty ) \end{array}$$

(25) $$\mathrm{Gumbel}:\;\begin{array}{c} C\left(u,v\right)=exp\left(-{\left({\left(-lnu\right)}^{\theta }+{\left(-lnv\right)}^{\theta }\right)}^{\frac{1}{\theta }}\right)\;\theta \in \left.1,\infty \right) \end{array}$$

where *C*(*u*,*v*) is the copula bivariate distribution function for marginal cumulative probabilities *u* and *v*. *θ* is the parameter which can be estimated by maximum likelihood method. 

The empirical copula function is introduced to estimate goodness of fit [46], which is written as:

(26) $$C_{\mathrm{emp}}\left(u,v\right)=\frac{1}{n}\sum_{i=1}^{n}I\left(\frac{R_{i}}{n+1}\leq u,\frac{S_{i}}{n+1}\leq v\right)$$

where *C_emp_*(*u*,*v*) is the empirical copula function. *R_i_* and *S_i_* are the ranks of the *i*th observed data. *I*(*A*) is the indicator function of event *A* which is presented as Equation (27).

(27) $$I\left(A\right)=\left\{\begin{array}{l} 1\;A\;is\;True\\ 0\;A\;is\;False \end{array}\right.$$

The fitted distributions were compared against empirical distribution and then the K-S tests and SED were used to test goodness of fit and select optimal copula function.

#### Bivariate Probability and Return Period

The bivariate probability and return period for *DD* vs. *DS*, *DD* vs. *DP* and *DS* vs. *DP* can be defined by two cases, namely Case “∩” and Case “∪”. Taking pair of *DD* vs. *DS* as an example, the two cases can be described as follow: 

(1) Case “∩”: (*DD* > *d*) and (*DS* > *s*)

(2) Case “∪”: (*DD* > *d*) or (*DS* > *s*)

The bivariate probabilities in these two cases can be estimated by Equations (28) and (29) [38].

(28) $$P_{D\cap S}=P\left(\begin{array}{ccc} DD\geq d & and & DS\geq s \end{array}\right)=1-F_{D}\left(d\right)-F_{S}\left(s\right)+C\left(F_{D}\left(d\right),F_{S}\left(s\right)\right)$$

(29) $$P_{D\cup S}=P\left(\begin{array}{ccc} DD\geq d & or & DS\geq s \end{array}\right)=1-C\left(F_{D}\left(d\right),F_{S}\left(s\right)\right)$$

where, *P_D_*_∩*P*_ and *P_D_*_∪*P*_ are bivariate probabilities for Case “∩” and Case “∪”, respectively. *F_D_*(·) and *F_S_*(·) are the marginal distributions for drought duration and severity, respectively. *C*(·) is the copula bivariate distribution for drought duration and severity. 

With the copula-based distribution, the bivariate return period in the mentioned two cases is denoted as [18,38]:

(30) $$T_{D\cap S}=\frac{\zeta }{P\left(\begin{array}{ccc} DD\geq d & and & DS\geq s \end{array}\right)}=\frac{\zeta }{1-F_{D}\left(d\right)-F_{S}\left(s\right)+C\left(F_{D}\left(d\right),F_{S}\left(s\right)\right)}$$

(31) $$T_{D\cup S}=\frac{\zeta }{P\left(\begin{array}{ccc} DD\geq d & or & DS\geq s \end{array}\right)}=\frac{\zeta }{1-C\left(F_{D}\left(d\right),F_{S}\left(s\right)\right)}$$

where, *T_D_*_∩*P*_ and *T_D_*_∪*P*_ are bivariate probabilities for Case “∩” and Case “∪”, respectively. *ζ* =*NY*/*ND*, *NY* refers to the period of SSDI time series (years), and *ND* is the number of drought events in *NY* years [39].

## 3. Results and Discussion

### 3.1. Time-Series Comparison of SSDI and SPEI with NDVI

**Figure 3:**
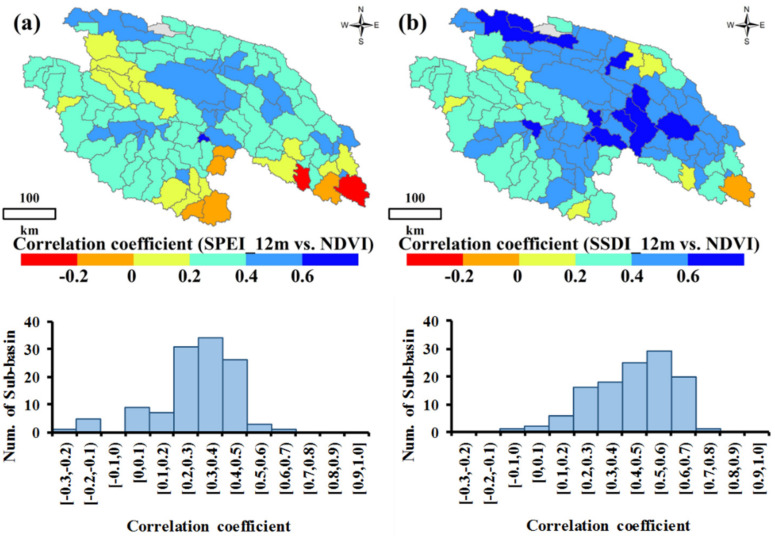
Drought event identification using the run theory for a given threshold level, SSDI = −1^*^: (**a**) the correlation coefficient between SPEI and NDVI; (**b**) the correlation coefficient between SSDI and NDVI; Note: sub-basins filled with grey mean there was no vegetation in these sub-basins; SSDI means standardized supply-demand water index.

One of the major motivations of this research is to develop a reliable and reasonable method that can be used to assess drought in forest and grass ecosystems objectively. The Normalized Difference Vegetation Index (NDVI) values can depict the vegetation biomass, which is an ancillary reflection of drought conditions [47]. In view of this dynamic characteristic, we compared the SSDI with NDVI to discover whether the new index constructed in this study can capture the regional drought. Figure 3 illustrates the relationship between SSDI/SPEI and NDVI during 2000–2015. It can be found that NDVI has much higher Pearson’s correlation coefficient with SSDI (*r_NDVI_*_, *SSDI*_) than does SPEI (*r_NDVI_*_, *SPEI*_). For example, the sub-basins with *r_NDVI_*_, *SSDI*_ ≥ 0.4 account for 63.4% of the total, while the sub-basins with *r_NDVI_*_, *SPEI*_ ≥ 0.4 account for only 25.6% (Figure 3). This indicates that the SSDI is superior to the SPEI for ecological drought assessment in the YRSR because it can capture the NDVI variation.

### 3.2. Temporal and Spatial Variability of Drought Characteristics

#### 3.2.1. Temporal Variability of Droughts

**Figure 4:**
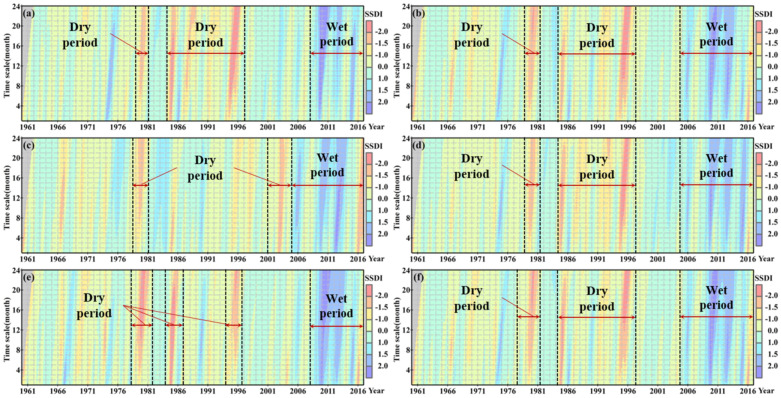
Hovmoller-type diagrams for the temporal variability of the SSDI from 1 to 24-month scales at different subregions: (**a**) Togton River Basin; (**b**) Middle stream; (**c**) Downstream; (**d**) Dam River Basin; (**e**) Qumar River Basin; (**f**) YRSR.

The interannual variability of the SSDI at different timescales is shown in Figure 4. We found that the SSDI series at different regions and the whole YRSR exhibit a wetting trend during 1960–2016. Taking the SSDI-12m of December as an example, the SSDI value increased by 0.0154 ± 0.0063 per year in the study period. It also can be seen that persistent droughts occurred frequently in 1977–1979 and 1984–1997, and periods of late-1970s, mid-1980s, and mid-1990s had witnessed the most severe drought episodes. A clear reversal of dry/wet condition in 2006 can be detected in Figure 3, which indicates that drought has been gradually relieved since mid-2000s. This change has happened largely because of the effects of climate change. A couple of studies have reported that the precipitation and actual evapotranspiration in the YRSR have been increased in the last decade [27,28,48]. In other words, the water supply for grassland and forest ecosystem would increase in this context, causing drought events to become less frequent in many regions of the YRSR.

**Figure 5:**
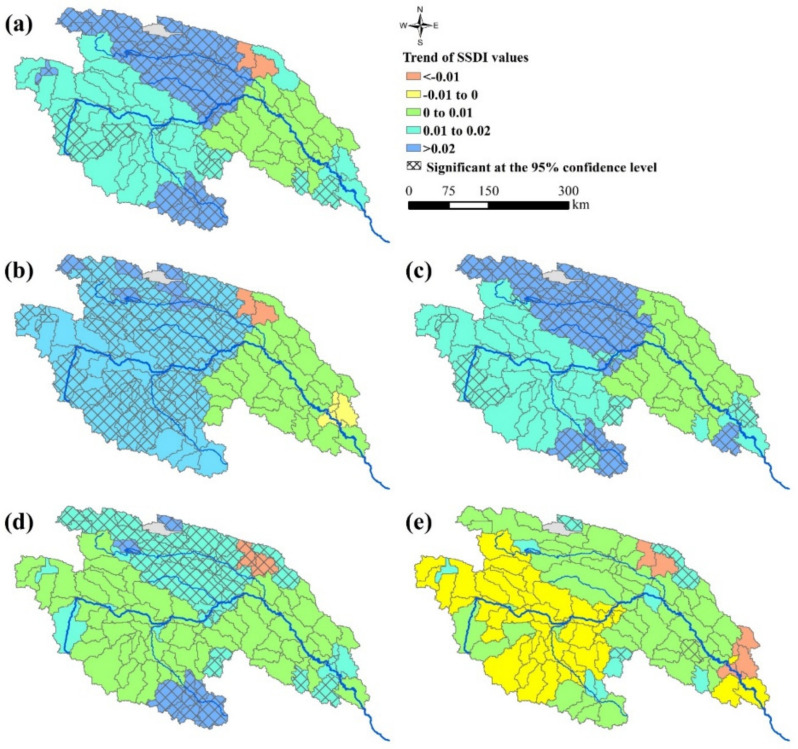
The trend of SSDI values during 1960–2016: (**a**) SSDI-12m of December; (**b**) SSDI-3m of May; (**c**) SSDI-3m of August; (**d**) SSDI-3m of November; (**e**) SSDI-3m of February. The crosshatch indicates that the trend is statistically significant at the 95% confidence level based on Mann–Kendall test.

To investigate the temporal evolution of dry/wet condition in YRSR, the linear regression analysis method and nonparametric Mann–Kendall test method were applied to analyze the trends of SSDI from 1960 to 2016. The dry/wet conditions of year, spring, summer, autumn, and winter are represented by SSDI-12m of December, SSDI-3m of May, SSDI-3m of August, SSDI-3m of November, and SSDI-3m of February (Figure 5). For more than four-fifths of the sub-basins across YRSR, the SSDI-12m of December presented increasing trends from 1960 to 2016 which indicate that most parts of YRSR have experienced wetting trends in the study period. In addition, the significant increasing trends of SSDI-12m (*α* = 0.05) have been detected in nearly half of the sub-basins, especially in northern YRSR (Figure 5a). The SSDI-3m of May, SSDI-3m of August, SSDI-3m of November showed a similar trend as the SSDI-12m, which illustrated that the dry conditions in spring, summer and autumn had been alleviated (Figure 5b–d). However, the change of SSDI values in winter was much different from that in other seasons. The dry condition in winter appeared to be more severe in Togton River Basin and Dam River Basin. However, the trend did not pass the significance test through the Mann–Kendall method (Figure 5e).

**Figure 6:**
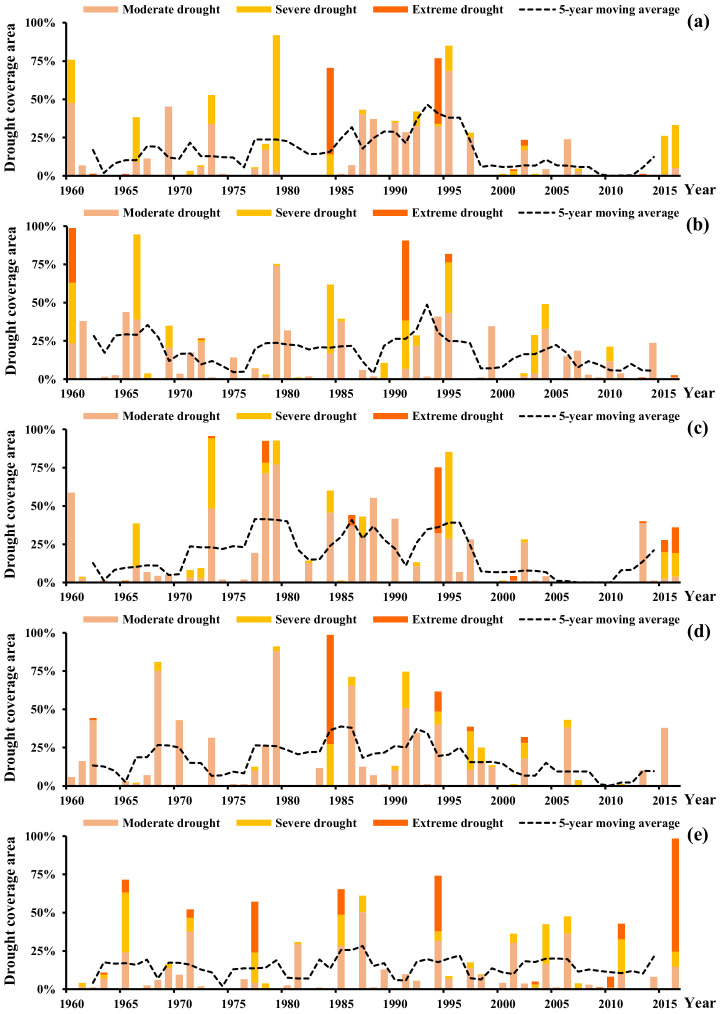
Percentages of drought affected areas for (**a**) year; (**b**) spring; (**c**) summer; (**d**) autumn; (**e**) winter.

Percentages of drought affected areas for seasonal and yearly scales were calculated with Equation (11) and the results are presented in Figure 6. It can be easily identified that the widespread drought events occurred frequently in the 1990s. The average percentages of drought affected areas (SSDI-12m < −1) were found to be 26.2% for this period, more than 1.6 times the average value for the 1960–2016 period. The notable severe ecological drought years were 1979, 1995, 1994, and 1960, with more than 75% of areas being affected (Figure 6a). The drought affected areas in spring, summer, and autumn have decreased since the early 2000s, but in winter have increased, especially in the most recent 10 years (Figure 6b–e). 

#### 3.2.2. Spatial Pattern of Drought Characteristics

**Figure 7:**
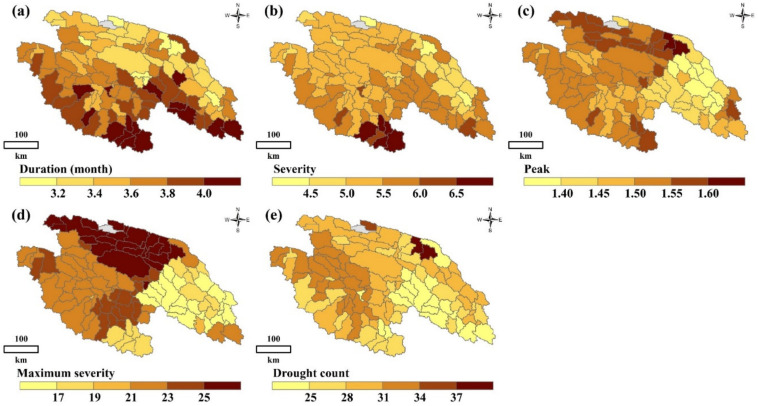
Spatial distribution of drought duration (**a**), severity (**b**), peak (**c**), maximum severity (**d**), and drought count (**e**) obtained on the basis of SSDI-6 values over YRSR during 1960–2016.

According to the drought event identification and characterization based on Run theory and SSDI at 6-month timescale, the spatial patterns of drought duration, severity, peak, maximum severity, and drought count in 1960–2016 are illustrated in Figure 7. The spatial distribution of average drought duration showed that the Southern YRSR (e.g., Dam River Basin, Nyaqa River Basin) suffered from droughts of a longer duration compared with the northern YRSR (e.g., Qumar River Basin, Beilu River Basin). However, the peak of SSDI in the northern YRSR was generally higher than that in Southern YRSR. The spatial distribution in average drought severity agreed well with that in drought duration, namely, high in the south and low in the north. In addition, the spatial distribution of maximum severity and number of drought events based on SSDI at 6-month timescale was computed in this research. It is clear that the higher maximum severity (*S_max_*) recorded by SSDI was within the northern YRSR, with *S_max_* more than 25. Average drought count was significantly higher in Togton River Basin and Dam River Basin, where precipitation is less and temperature is lower. 

#### 3.2.3. Effect of Time Scales on Drought Characteristics

**Figure 8:**
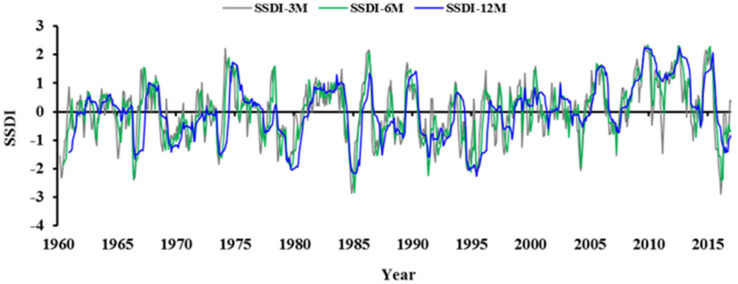
Time series of the 3, 6, and 12 months SSDI of the YRSR for the period 1960–2016.

**Figure 9:**
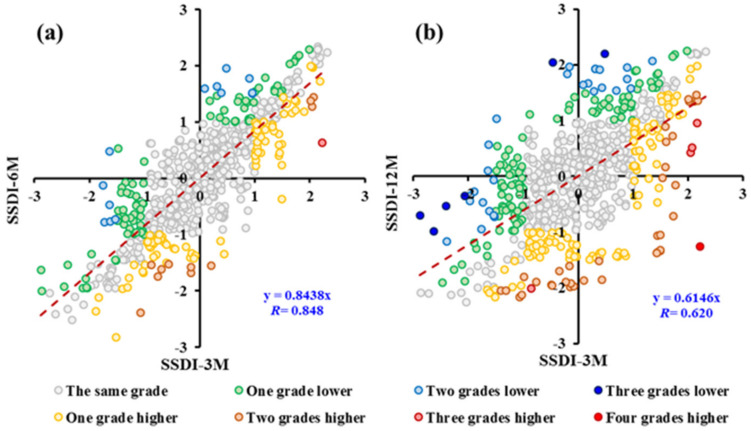
Scatter plot between (**a**) SSDI-3M and SSDI-6M; (**b**) SSDI-3M and SSDI-12M.

**Figure 10:**
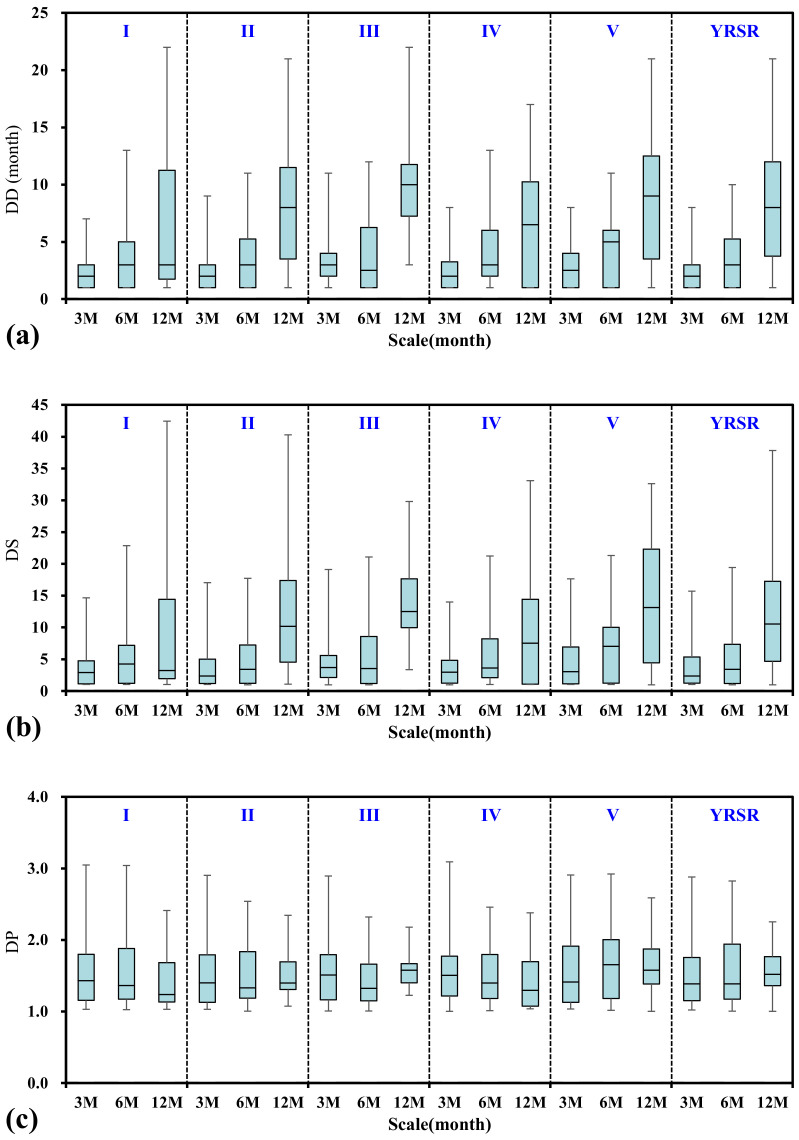
The box-whisker plots for drought characteristics: (**a**) drought duration, (**b**) drought severity, and (**c**) drought peak. I–V represent Togton River Basin, Middle stream, Downstream, Dam River Basin, and Qumar River Basin, respectively.

The time scale of SSDI is flexible, e.g., a 3-month SSDI can be used as a short-term drought index while a 12-month SSDI can be used as an intermediate-term one. Figure 8 compared the SSDI value for the time scales of 3, 6, and 12 months. It can be found that the time series of SSDI-12M are more ‘smoothed’ than that of SSDI-3M and SSDI-6M. That is, the dry/wet conditions identified by smaller time scale SSDI took place more alternately. The scatter plots for SSDI-3M and SSDI-6M/12M are shown in Figure 9. Drought grades identified by SSDI-6M/12M were generally lower than that identified by SSDI-3M. The difference between SSDI-3M and SSDI-6M was relatively smaller with the correlation coefficient nearly to 0.85. However, the difference between SSDI-3M and SSDI-12M was obvious. The characteristics of drought events derived from multiscale SSDI were compared in Figure 10. It can be concluded that the drought characteristics are affected by the time scales. The drought event appears to be longer and intensified but low frequency at higher timescales. Taking the entire YRSR as an example, the median drought duration and severity were 2.0 months and 2.4, respectively, at 3-month scale, while 8.0 months and 10.5, respectively, at 12-month scale. These results show that the choice of time scales to calculate SSDI value can introduce uncertainties in drought characteristics evaluated. 

#### 3.2.4. Sensitivity of Drought Characteristics to the Temperature

**Figure 11:**
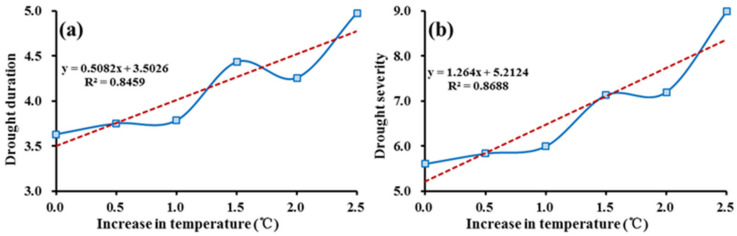
Sensitivity on average (**a**) drought duration (*DD*) and (**b**) severity (*DS*) due to temperature change in the YRSR.

**Figure 12:**
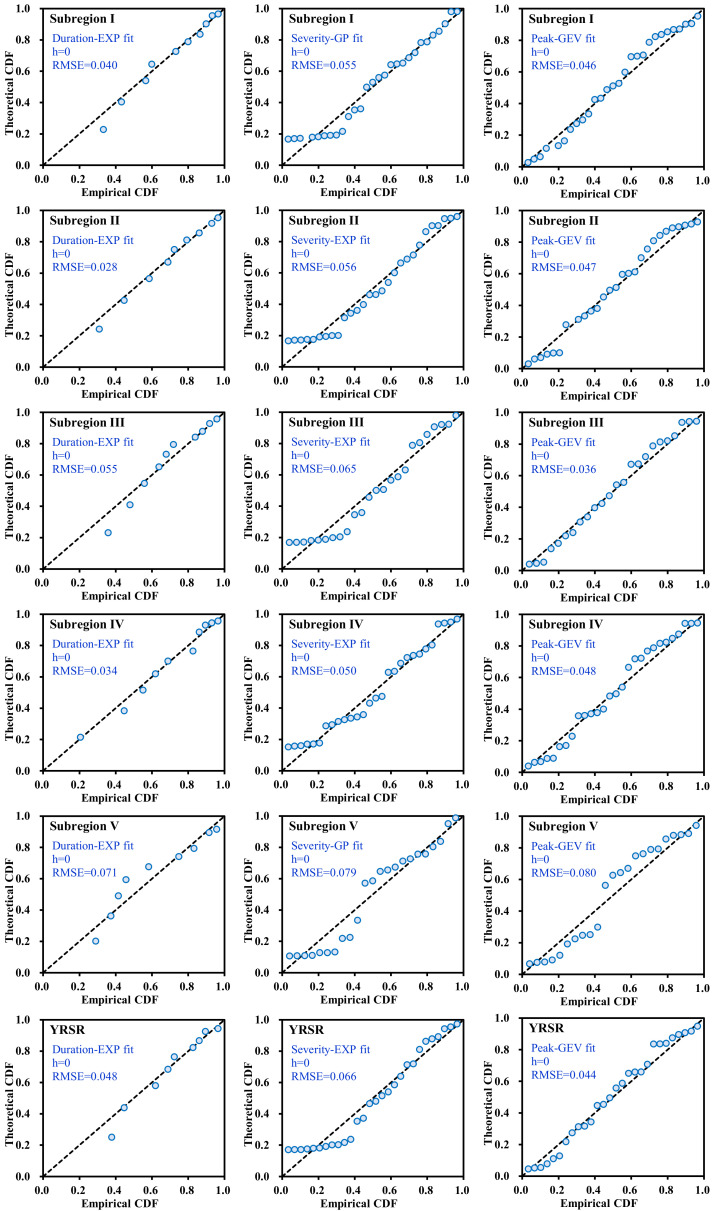
Comparison between empirical and theoretical CDFs of drought duration, severity, and peak in subregions I–V and entire YRSR. EXP, WBL, GP, GEV, and GAM are abbreviations for Exponential, Weibull, Generalized Pareto, Gamma, and Generalized Extreme Value distributions. The value of *h* is 1 if the test rejects the null hypothesis at the 5% significance level, or 0 otherwise. RMSE is the value of root-mean-square error. The dotted line indicates the 1:1 line.

Previous studies have shown that the temperature has been increasing significantly in the YRSR [49,50,51]. In the context of warming climate, both the green water flow and ecological water requirement would increase, which may lead to complex changes in the balance between water supply and demand. Additionally, then drought characteristics are likely to be changed accordingly. To investigate the sensitivity of drought characteristics to the temperature in the YRSR, a series of hypothetical temperature increase scenarios, changing from 0.5 to 2.5 °C in increments of 0.5 °C, were simulated and analyzed. Generally, the drought duration and severity in the whole YRSR would increase along with the increase of temperature (Figure 11). In the hypothetical scenarios with Δ*T* ≤ 1 °C, the average drought duration and severity were less susceptible to changes in temperature. The resulting slope indicates that a 1 °C increase in temperature corresponds to increases in average drought duration and severity by 4.2% and 6.9%, respectively (dashed red lines in Figure 11). It is clear that the drought characteristics were more susceptible to the temperature increase in the hypothetical scenarios with Δ*T* > 1 °C. The results showed increases of 20.7% and 32.6% for drought duration and severity respectively when temperature was increased by 1 °C (dashed red lines in Figure 12). According to the previous study, the annual average temperature in the YRSR was projected to increase by 2.2 °C in the next 30 years based on CMIP5 Climate Models [28]. In addition, that means the ecological droughts with longer duration and larger severity may happen more frequently in the future. However, this trend needs to be further explored because the precipitation was also projected to increase. In addition, the temperature increase would also have an effect on growth period of grass or forest in the YRSR [51,52,53]. This change in phenology would alter the water demand process and drought characteristics indirectly, which should be further investigated.

### 3.3. Regional Bivariate Probability and Return Period

#### 3.3.1. Selection of the Marginal Distributions and Copulas

The exponential, Gamma, Weibull, Generalized Pareto, and Generalized Extreme Value distributions were chosen as appropriate marginal distributions for the *DD*, *DS*, and *DP* in each subregion and the entire YRSR. The best fit distributions for each drought variable were selected. The theoretical probability and empirical probability of selected distribution for drought variables were plotted in Figure 12. It can be seen from Figure 12 that the associations of theoretical and empirical probabilities are close to the 1:1 line, which proved the reliability of marginal distribution selection. 

**Figure 13:**
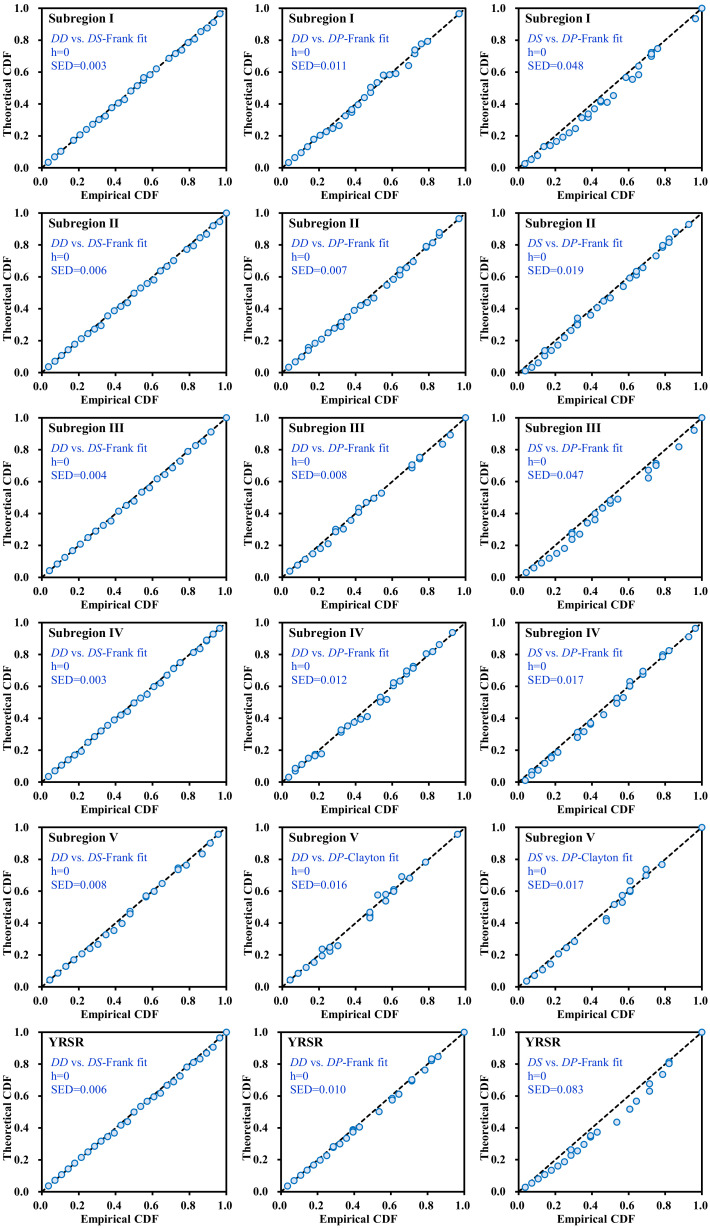
Comparison between empirical and theoretical CDFs of bivariate distribution in subregions I–V and entire YRSR. The value of *h* is 1 if the test rejects the null hypothesis at the 5% significance level, or 0 otherwise. SED is the value of square Euclidean distance. The dotted line indicates the 1:1 line.

With the optimal marginal distribution of drought variables chosen above, Copula function for modeling bivariate distribution could be structured. Three kinds of Archimedean copulas, namely Clayton, Frank and Gumbel, were compared in this research and the estimated parameters for best-fitted copulas are listed in Table 2. In case of *DD* vs. *DS*, the Frank Copula in all six subregions and the entire YRSR was the best function with the lowest SED value. Details of the optimal copulas for *DD* vs. *DP* and *DS* vs. *DP* in subregions can be found in Table 2. The comparison plot between theoretical joint cumulative probability and empirical joint cumulative probability is shown in Figure 13. It could be observed that the theoretical value is almost equal to the empirical one. This result proved that the selected copulas and estimated parameters are reliable. It should be noted that Frank Copula appears to be proper and robust in modeling bivariate distribution of drought variables in many of the subregions of YRSR.

#### 3.3.2. Regional Bivariate Probability of Drought Events

**Figure 14:**
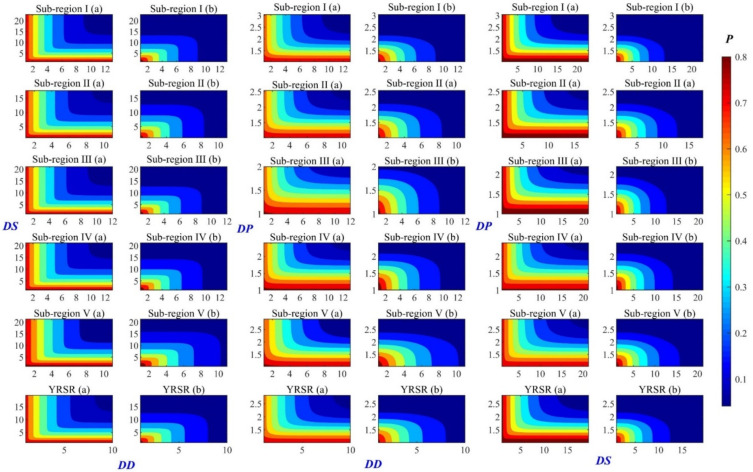
Bivariate probability of *DD* vs. *DS*, *DD* vs. *DP* and *DS* vs. *DP*: (a) “∪” bivariate probability; (b) “∩” bivariate probability.

Using the selected optimal copulas in I–V subregions and the entire YRSR, the bivariate probability of *DD* vs. *DS, DD* vs. *DP* and *DS* vs. *DP* in both Case “∩” and Case “∪” can be estimated and the results are presented in Figure 14. For example, when the *DD* and *DS* exceed 3 months and 3, respectively (above moderate grade), the *P_D_*_∩*S*_ was 0.459, 0.434, 0.453, 0.484, 0.508 and the *P_D_*_∪*S*_ was 0.590, 0.580, 0.577, 0.613, 0.645 in subregions I–V, respectively. For the drought event with *DD* > 3 and *DS* > 4.5(above severe grade), the *P_D_*_∩*S*_ in the five subregions was 0.435, 0.411, 0.422, 0.461, 0.489 and *P_D_*_∪*S*_ was 0.478, 0.465, 0.467, 0.504, 0.537. It could be found that the joint probabilities are different among the five subregions, with significantly larger *P_D_*_∩*S*_ and *P_D_*_∪*S*_ in Dam River Basin and Qumar River Basin, implying that the drought risk is higher in these regions.

#### 3.3.3. Bivariate Return Period of Drought Events

**Figure 15:**
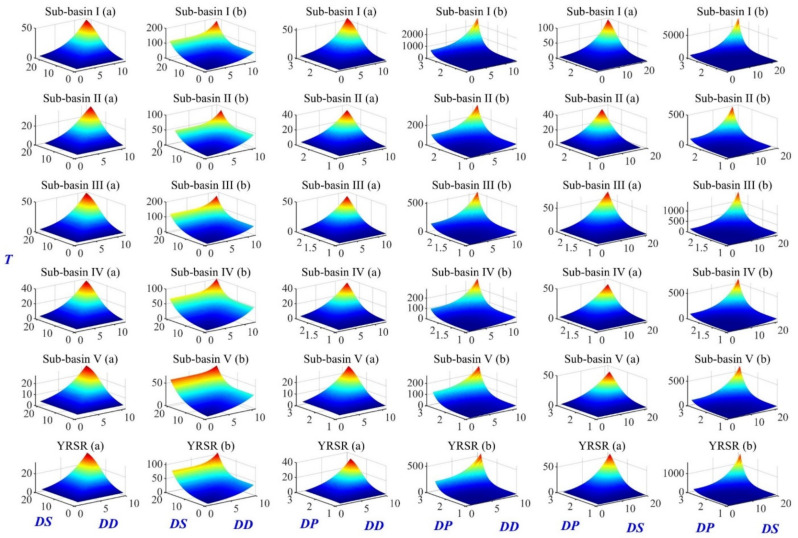
Bivariate return periods of *DD* vs. *DS*, *DD* vs. *DP* and *DS* vs. *DP*: (a) “∪” return period; (b) “∩” return period. *DD*: Drought duration; *DS*: Drought severity; *DP*: Drought peak.

The bivariate return periods (*T*) of *DD* vs. *DS*, *DD* vs. *DP* and *DS* vs. *DP* for subregions I–V and the YRSR were estimated according to Equations (30) and (31), and results are illustrated in Figure 15. It can be found that various pairs of drought variables can result in the same return period. With the 3D-surface figure, the associated return period in Case ∩ and Case ∪ for a specific drought variable can be extracted. For example, *DD* = 6 months and *DS* = 6 result in *T_D_*_∩*S*_ = 11.5 year and *T_D_*_∪*S*_ = 6.2 years for the YRSR. The two kinds of return period are compared, and the “∪” return periods are shorter than the “∩” return periods in all the sub-basins and the entire YRSR. Further, the univariate and bivariate drought return periods for different variation patterns were compared (Table 3). Apparently, the univariate return period is longer than “∪” return periods, while shorter than the “∩” return periods. With the *DD*, *DP* and *DS* increasing, both the “∪” and “∩” return periods increased. In addition, the difference between “∪” and “∩” return periods would also increase as the values of drought variables increase. Taking the entire YRSR as an example, the *DD*, *DS* and *DP* were about 11.1, 17.2 and 1.8, respectively, in a situation whereby the univariate return period was 50 years. For this particular event, the *T_D_*_∪*S*_, *T_D_*_∪*S*_ and *T_P_*_∪*S*_ were about 34.1, 28.9 and 29.6 years while the *T_D_*_∩*S*_, *T_D_*_∩*S*_ and *T_P_*_∩*S*_ were about 93.3, 186.6 and 161.5 years. The results are important for drought risk assessment and would be helpful in planning and management of water resources systems under severe or extreme drought scenarios.

### 3.4. Spatial Distribution of Bivariate Probability and Return Period in the YRSR

**Figure 16:**
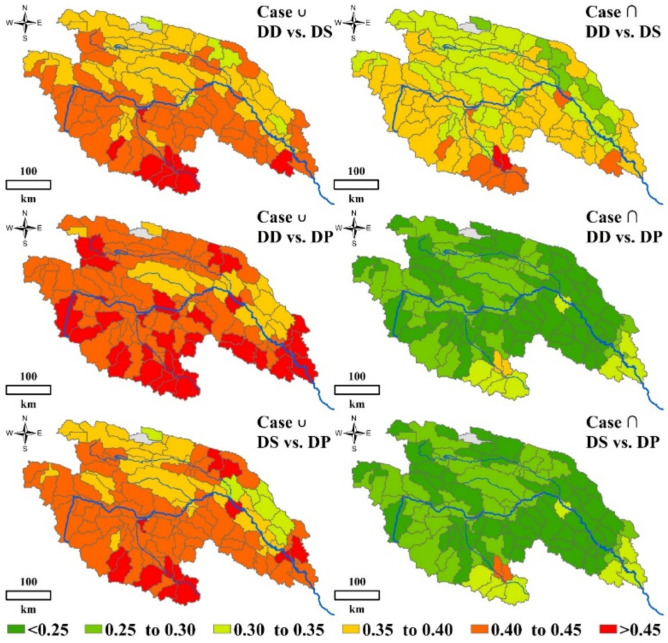
Spatial distribution of bivariate probabilities of *DD* vs. *DS*, *DD* vs. *DP* and *DS* vs. *DP* in Case ∪ and Case ∩ in the YRSR.

The average *DD*, *DS* and *DP* for the YRSR are 3.46 months, 5.37 and 1.54, respectively. For these particular drought characteristics, the bivariate probabilities of the 119 sub-basins divided for spatial analysis [28] were estimated and mapped in Figure 16. A noticeable spatial variability of *P_D_*_∪*S*_ and *P_D_*_∩*S*_ could be observed obviously. The bivariate probabilities of *DD* vs. *DS* increased from the north to the south, implying that the droughts in southern YRSR are relatively more severe and have longer duration. In the case of *DD* > 3.46 and *DS* > 5.37, the bivariate probabilities of *P_D_*_∪*S*_ and *P_D_*_∩*S*_ were more than 0.40 and more than 0.35 in the Dam River Basin. While in the Qumar River Basin, *P_D_*_∪*S*_ and *P_D_*_∩*S*_ were evaluated as 0.30–0.45 and 0.25–0.40, respectively. Comparing with *P_D_*_∪*S*_ and *P_D_*_∩*S*_, the spatial difference of *P_D_*_∪*P*_, *P_D_*_∩*P*_, *P_S_*_∪*P*_, and *P_S_*_∩*P*_ among YRSR is not obvious. *P_D_*_∪*P*_ and *P_S_*_∪*P*_ in nearly all of the sub-basins in YRSR were more than 0.45. The *P_D_*_∩*P*_ and *P_S_*_∩*P*_ in most parts of YRSR ranged from 0.30 to 0.33.

**Figure 17:**
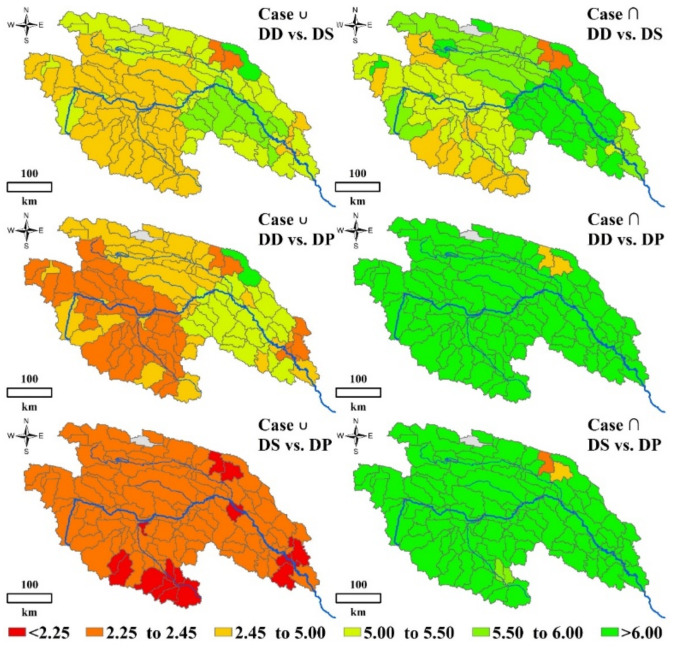
Spatial distribution of bivariate return periods of *DD* vs. *DS*, *DD* vs. *DP* and *DS* vs. *DP* in Case ∪ and Case ∩ in the YRSR.

The spatial distribution of bivariate return periods is similar with that of probabilities (Figure 17). The short return periods are often associated with high probabilities and dispersed in southern YRSR, especially for *DD* vs. *DS*. In most areas of Togton River Basin and Dam River Basin, the *T_D_*_∪*S*_ and *T_D_*_∩*S*_ remained in the variation range from 2.33–2.49 years and 2.67–2.85 years. While, those two kinds of return periods in Qumar River Basin were around 2.6 and 3.0 years, respectively. These results suggest that severe ecological drought events are more likely to occur in the Togton River Basin and Dam River Basin.

## 4. Conclusions

This study introduced a new natural vegetation drought index, standardized supply-demand water index (SSDI), and identified spatial-temporal variability of natural vegetation drought characteristics drought events. Then the drought-prone regions were investigated. The primary conclusions are as follows:

1. The time-series of SSDI and Standardized Precipitation and Evapotranspiration Drought Index (SPEI) with Normalized Difference Vegetation Index (NDVI) were compared in this study. There exists a higher correlation between constructed SSDI and NDVI. This result indicated that the constructed SSDI was reliable and can reflect the comprehensive characteristics of the ecological drought information more easily and effectively.
2. The YRSR had witnessed the most severe drought episodes in the periods of late-1970s, mid-1980s and mid-1990s, but the SSDI showed a wetting trend since the mid-2000s, mainly because of a warmer and wetter climate in the most recent 10 years. However, the climate change has different effects on the dry condition at seasonal scales. The drought affected areas in spring, summer and autumn have decreased since 2000 while this area in winter has increased. The drought duration and severity showed a spatial variation among different regions in the YRSR. Generally, droughts in the Southern YRSR were relatively more severe with longer drought duration, implying that the Southern YRSR was an area that had been facing challenging drought conditions. The average drought duration and severity in the YRSR would be less susceptible to changes in temperature when the increase temperature was above 1.0 °C. However, the characteristics would be more susceptible to temperature in the YRSR when the increase temperature were above 1.0 °C. The average drought duration and severity is shown to increase by 20.7% and 32.6% with a 1 °C increase in temperature for the hypothetical scenarios with Δ*T* > 1 °C.
3. The return periods of five sub-basins and the entire YRSR for case ‘‘∩” were longer than those in case ‘‘∪” and their spatial trends are highly consistent. High return periods were found in Qumar River Basin. While, low return periods were found in most areas of Togton River Basin and Dam River Basin, implying that severe ecological drought events occurred more frequently.

The study investigated the spatial-temporal variation characterization of ecological drought in the Yangtze River Source Region, which provides a comprehensive understanding of regional drought events in the YRSR and highlights the bivariate drought probabilities and return periods. However, two aspects should be improved in further study. First, the choice of time scales to calculate SSDI value can introduce uncertainties in drought characteristics evaluated. Therefore, future study should compare multiscale SSDI indices and select the optimal time scale for drought assessment in the YRSR. Second, temperature is one of the most crucial and direct driving factors of the changes in growth period. Additionally, the changes in growth period for grass and forest could affect the process of ecological water requirement. This dynamic change should be quantified in the algorithm of SSDI. Furthermore, the current paper selected the YRSR as a case study and the methodological development of SSDI and drought identification can be used in other catchments or regions as well.

## Figures and Tables

**Table 1:** Classification of SSDI (standardized supply-demand water index) and drought grade.

Drought Grade	Range of *SSDI* Value
Near normal	1.00 > *SSDI* > −1.00
Moderate drought	−1.00 ≥ *SSDI* > −1.50
Severe drought	−1.50 ≥ *SSDI* > −2.00
Extreme drought	−2.00 ≥ *SSDI*

**Table 2:** The estimated parameters of the best-fitted copulas for subregions I–V and entire YRSR.

Subregion	*DD* vs. *DS*		*DD* vs. *DP*		*DS* vs. *DP*	
	Copula	Parameter (*θ*)	Copula	Parameter (*θ*)	Copula	Parameter (*θ*)
I	Frank	33.094	Frank	8.192	Frank	10.202
II	Frank	25.537	Frank	9.184	Frank	11.220
III	Frank	31.090	Frank	7.205	Frank	8.897
IV	Frank	32.708	Frank	9.804	Frank	11.906
V	Frank	18.411	Clayton	2.042	Clayton	3.509
YRSR	Frank	27.058	Frank	9.024	Frank	10.889

**Table 3:** The bivariate return period (years) of *DD* vs. *DS*, *DD* vs. *DP* and *DS* vs. *DP.*

Region	*T*	*DD*	*DS*	*DP*	*DD* vs. *DS* Return Period		*DD* vs. *DP* Return Period		*DS* vs. *DP* Return Period	
					Case∪	Case ∩	Case∪	Case ∩	Case∪	Case ∩
Subregion I	5	3.6	5.3	0.2	4.7	5.3	4.1	6.3	4.3	6.0
	10	6.3	9.2	0.6	9.0	11.2	7.3	15.7	7.6	14.5
	20	9.0	13.2	1.0	16.5	25.4	12.9	44.1	13.4	39.2
	50	12.5	18.4	2.0	35.1	87.1	28.5	204.2	29.2	173.7
	100	15.2	22.3	3.1	62.3	253.9	53.7	719.7	54.6	598.3
Subregion II	5	3.2	4.9	0.2	4.7	5.4	4.2	6.1	4.3	5.9
	10	5.7	8.7	0.5	8.8	11.5	7.5	14.9	7.8	13.9
	20	8.2	12.6	1.0	16.0	26.7	13.3	40.6	13.7	36.7
	50	11.5	17.6	1.9	33.8	96.1	29.0	182.6	29.7	158.2
	100	14.0	21.4	3.0	60.4	289.5	54.3	633.5	55.1	536.3
Subregion III	5	2.8	4.1	0.1	4.8	5.2	4.2	6.2	4.3	6.0
	10	5.5	7.8	0.4	9.1	11.0	7.4	15.4	7.7	14.3
	20	8.1	11.6	0.7	16.9	24.5	13.1	42.6	13.6	38.1
	50	11.6	16.6	1.2	36.0	81.6	28.7	194.9	29.4	167.1
	100	14.2	20.4	1.7	63.7	232.0	54.0	682.7	54.8	571.7
Subregion IV	5	3.7	5.5	0.2	4.8	5.3	4.3	6.0	4.4	5.8
	10	6.6	9.8	0.5	9.1	11.2	7.6	14.5	7.9	13.6
	20	9.5	14.0	0.8	16.6	25.1	13.4	39.2	13.9	35.7
	50	13.3	19.6	1.3	35.4	85.4	29.2	174.1	29.9	151.8
	100	16.1	23.9	1.8	62.7	246.9	54.5	599.6	55.4	511.0
Subregion V	5	3.1	4.8	0.1	4.8	5.3	4.3	5.9	4.5	5.6
	10	6.2	9.5	0.6	9.1	11.1	7.7	14.2	8.2	12.8
	20	9.3	14.3	1.0	16.8	24.6	13.6	38.0	14.5	32.1
	50	13.3	20.5	1.7	35.9	82.2	29.4	166.1	31.0	129.6
	100	16.4	25.3	2.4	63.6	234.5	54.8	567.8	56.7	422.9
YRSR	5	3.1	4.8	0.2	4.7	5.3	4.2	6.1	4.3	5.9
	10	5.5	8.5	0.6	8.9	11.4	7.5	15.0	7.8	14.0
	20	7.9	12.3	1.0	16.1	26.3	13.2	41.0	13.7	37.2
	50	11.1	17.2	1.8	34.1	93.3	28.9	185.0	29.6	161.5
	100	13.5	20.9	2.7	60.9	278.6	54.2	643.0	55.0	549.6

Note: The first column is the given return period of single variable DD, DS and DP. The second to the fourth columns are the value of DD, DS and DP with given return period, which can be obtained by inverse function of Equations (16)–(20). The sixth to eleventh column are the combined and co-occurrence return period, which can be obtained by Equations (30)–(31).
